# HIV-1 Tropism and Liver Fibrosis in HIV–HCV Co-Infected Patients

**DOI:** 10.1371/journal.pone.0050289

**Published:** 2012-11-30

**Authors:** Florence Abravanel, Stéphanie Raymond, Elodie Pambrun, Maria Winnock, Philippe Bonnard, Philippe Sogni, Pascale Trimoulet, François Dabis, Dominique Salmon-Ceron, Jacques Izopet, ANRS CO13 HEPAVIH Study Group

**Affiliations:** 1 INSERM, U1043, Centre de Physiopathologie de Toulouse Purpan, Toulouse, France; 2 CHU Toulouse Purpan, Laboratoire de Virologie, Institut Fédératif de Biologie de Purpan, France; 3 INSERM, U897 and ISPED, Université Victor Segalen, Bordeaux, France; 4 Service des Maladies Infectieuses et Tropicales, Hôpital Tenon, AP-HP, Paris, France; 5 Unité d'Hépatologie, Hôpital Cochin, AP-HP, Paris, France; 6 Université Paris Descartes, Paris, France; 7 CHU Pellegrin, Laboratoire de virologie, Bordeaux, France; 8 CNRS, UMR 5234, Microbiologie fondamentale et pathogénicité, Université Bordeaux Segalen, Bordeaux, France; 9 Service des Maladies Infectieuses et Tropicales, Hôpital Cochin, AP-HP, Paris, France; National Institute of Allergy and Infectious Diseases, United States of America

## Abstract

**Background and Aims:**

Hepatic stellate cells, the major producers of extracellular matrix in the liver, and hepatocytes bear CXCR4 and CCR5, the two main co-receptors for entry of the human immunodeficiency virus (HIV). *In vitro* studies suggest that HIV-envelope proteins can modulate the replication of hepatitis C virus (HCV) and fibrogenesis. We investigated the influence of HIV tropism on liver fibrosis and the concentration of HCV RNA in HIV–HCV co-infected patients.

**Methods:**

We used a phenotypic assay to assess HIV tropism in 172 HCV–HIV co-infected patients: one group (75 patients) had mild fibrosis (score ≤F2) and the other (97 patients) had severe fibrosis (score >F2). We also assessed the relationship between HIV tropism and HCV RNA concentration in all these patients. We also followed 34 of these patients for 3 years to determine the evolution of HIV tropism and liver fibrosis, estimated by liver stiffness.

**Results:**

Initially, most patients (91.8%) received a potent antiretroviral therapy. CXCR4-using viruses were found in 29% of patients. The only factor associated with a CXCR4-using virus infection in multivariate analysis was the nadir of CD4 cells: <200/mm^3^ (OR: 3.94, 95%CI: 1.39–11.14). The median HCV RNA concentrations in patients infected with R5 viruses, those with dual-mixed viruses and those with X4 viruses, were all similar. The prevalence of CXCR4-using viruses in patients with mild fibrosis (≤F2) (31%) and those with severe fibrosis (F3–F4) (28%, *p* = 0.6) was similar. Longitudinal analyses showed that the presence of CXCR4-using viruses did not increase the likelihood of fibrosis progression, evaluated by measuring liver stiffness.

**Conclusions:**

The presence of CXCR4-using viruses in patients receiving a potent antiretroviral therapy does not influence HCV RNA concentration or liver fibrosis.

## Introduction

Liver disease has emerged as a leading cause of death among people in Europe and the United States infected with the human immunodeficiency virus (HIV), and most of these deaths are caused by chronic viral hepatitis [1]. Co-infection with hepatitis C virus (HCV) and HIV is common because these viruses share the same transmission route. HIV infection modifies the natural course of HCV infection in several ways [2], [3], [4], [5]. HCV RNA concentrations are increased in HIV-infected patients [6]. Infection with HIV enhances HCV transmission, particularly mother-to-child transmission [7], and decreases the rates of spontaneous HCV clearance, which leads to higher rates of chronic HCV infection [6]. Lastly, liver disease progresses more rapidly in HIV–HCV co-infected patients than in patients infected with HCV alone [3], [8], [9]. However, the mechanisms by which HIV infection increase the risk of liver disease are poorly understood and are probably multi-factorial [2].

HIV enters CD4-expressing cells using one or both of the chemokine receptors: C–C chemokine receptor type 5 (CCR5) and C–X–C chemokine receptor type 4 (CXCR4). CCR5-using viruses are classified as R5 variants, CXCR4-using viruses are classified as X4 variants, and viruses that can use both co-receptors are classified as dual-mixed variants (D/M) [10]. Hepatic stellate cells (HSCs), the major producers of the extracellular matrix in the liver [11], bear functional CXCR4 and CCR5 receptors [12], [13]. Primary hepatocytes also bear both co-receptors and can be infected by HIV [14].

*In vitro* studies suggest that the HIV gp120 protein modulates fibrogenesis and the concentration of HCV RNA in HIV–HCV co-infected patients [15], [16], [17], [18], [19]. HIV increases the replication of HCV in the JFH1 model and in HCV subgenomic replicons *in vitro* [16]. This increase is mediated by the interaction of the HIV-envelope glycoprotein, gp120, with CCR5 and CXCR4, and depends on transforming growth factor beta 1 [16]. The HSCs respond to exposure to CCR5-tropic recombinant gp120 by increasing chemotaxis and expression of genes that encode for the proinflammatory chemokine, the monocyte chemoattractant protein-1, interleukin 6 and the tissue inhibitor of metalloprotease-1 [15]: however, collagen production increases only slightly [15]. In contrast, HSCs infected with the X4 virus increase collagen synthesis and secrete more monocyte chemoattractant protein-1 [17].

CXCR4-tropic recombinant gp120 promotes the synthesis of fibrogenic markers in HSCs [20]. Other studies using various recombinant gp120 proteins or entire HIV virions with different types of tropism indicate that HIV enhances apoptosis of hepatocytes, which is mediated by the CXCR4 receptor [18], [19]. The HCV-envelope proteins, E2, act in cooperation with CXCR4-tropic gp120 HIV-envelope proteins to trigger apoptosis in hepatocytes via an innocent bystander mechanism [21]. There is also evidence that the phagocytotic clearance of apoptotic debris may directly stimulate fibrogenesis [22], [23], [24]. Thus, there seems to be a direct pathway linking HIV infection with liver fibrogenesis via the envelope proteins.

We analysed these *in vitro* data and developed a working hypothesis that CXCR4-using viruses are more pathogenic in terms of liver damage than the R5 virus. We then investigated the influence of HIV tropism on liver fibrosis by assessing the prevalence of CXCR4-using viruses in two groups of HCV–HIV co-infected patients: one with mild fibrosis (score ≤F2) and the other with severe fibrosis (score >F2). We also followed 34 patients for 3 years and assessed the influences of HIV tropism on fibrosis progression by studying liver stiffness. Lastly, we assessed the influence of HIV-1 tropism on the plasma concentration of HCV RNA.

## Patients and Methods

### Participants

Patients were enrolled from the ANRS CO13 HEPAVIH nationwide cohort of HIV–HCV co-infected individuals [25]. They all had HIV-1 antibodies and a chronic HCV infection that had been confirmed by Western blotting and plasma HCV-RNA assays. Patients who agreed to participate gave their written informed consent.

Based on the *in vitro* studies, we postulated that CXCR4-using viruses have a more deleterious impact on liver disease than CCR5-using viruses. We calculated the sample size required in a cross-sectional study, which detected a 20% difference in the prevalence of CXCR4-using viruses between patients with mild fibrosis (score: F0–F2) and those with severe fibrosis (score: F3–F4) using a one-sided test, with 80% power and a 0.05 alpha risk. According to several studies, the prevalence of CXCR4-using viruses in HIV patients at similar stages of infection is estimated to be approximately 30% [26], [27]. Thus, if the prevalence of CXCR4-using viruses is 30% in patients with mild fibrosis, each group should contain 73 patients in order to detect a 20% difference in prevalence (one-sided test, 80% power, 0.05 alpha risk). Thus, we recruited a total of 195 patients: 90 with mild fibrosis and 105 with severe fibrosis.

These patients met the following inclusion criteria: patients had either had a liver biopsy the year before inclusion or a clinician has confirmed liver cirrhosis at inclusion, they had a blood sample available at inclusion, and their dates of HCV and HIV acquisition were known. We estimated the date of HCV infection to be 1 year after the onset of being an intravenous drug user (IVDU), whereas the date of HIV infection was based on the first positive serological test. The day of inclusion into the cohort, and the clinical and biological data, were entered into a clinical research form completed by the medical staff at the respective clinical centres.

HIV tropism was determined using a phenotypic assay in 172 patients (75 with mild fibrosis, 97 with a fibrosis score of F3–F4). We failed to determine HIV tropism in 23 patients for technical reasons. In 34 of these patients, we also determined the evolution of HIV tropism and liver fibrosis, estimated by measuring liver stiffness, after a median period of 36 months (IQR: 35–36 months).

### Liver fibrosis

Most (103) patients underwent a liver biopsy the year before their inclusion. The grade and stage of chronic hepatitis were assessed in the liver biopsies according to the Metavir classification [28]. The remaining 69 patients were classified as having a fibrosis score of F4 because their clinical signs of cirrhosis were verified by the examining clinician (oesophageal varices, ascites, liver encephalopathy or intestinal bleeds). We also used transient elastography with a Fibroscan machine (EchoSens) to determine liver stiffness at inclusion and after a median period of 36 months (inter-quartile range [IQR]: 35–36 months) in the 34 patients followed-up long term [29].

### HIV tropism

Blood samples were collected on the day of inclusion. A recombinant virus phenotypic entry assay was used to determine HIV-1 co-receptor usage [30], [31], [32]. This test is suitable for use with blood plasma and cell samples. A fragment encompassing the gp120 and ectodomain of gp41 was amplified from HIV RNA in the plasma by RT-PCR, or from HIV DNA within a whole-blood sample using PCR. HIV-1 tropism was determined by amplifying HIV DNA in the cells of 145 patients whose HIV RNA was ≤400 copies/mL and by amplifying the HIV RNA from 27 patients whose plasma HIV RNA was >400 copies/mL. HIV tropism was also determined from HIV DNA in blood samples from the 34 long-term follow-up patients collected 36 months after their inclusion.

### Plasma HCV RNA

We determined the concentration of HCV RNA in the plasma of blood samples collected at cohort inception using the Cobas Ampliprep-Cobas Taqman test (Roche Diagnostics, Meylan, France).

### Statistical analyses

Descriptive values are expressed as medians with their IQRs. Non-parametric tests were used to compare the differences between the groups (Wilcoxon test for continuous variable, χ^2^ test for qualitative variables). A *p*-value of <0.05 was considered significant. Baseline predictors of CXCR4 tropism were evaluated by univariate and multivariate analyses.

The following covariates were analysed at inclusion: age, gender, age at HCV and HIV infections, duration of HCV and HIV infections, source of HCV and HIV infections, liver steatosis, CD4-cell count at inclusion, nadir of CD4-cell count, type of antiretroviral therapy, but also the type and duration of any previous antiretroviral therapy before inclusion into the cohort, HCV genotype, HCV RNA plasma concentration, and plasma HIV RNA concentration. Variables with a *p*-value of ≤0.25 after univariate analyses were entered into multivariate, backward, stepwise logistic regression analyses to identify significant variables independently associated with the presence of CXCR4-using viruses. Odds ratios (OR) were estimated from the model and are given with their 95% confidence intervals (CI).

The same methods and covariates, including HIV tropism (classified as CCR5 or CXCR4-using viruses), geographic origin of the patients, body-mass index, alcohol consumption in the past or at cohort inception, insulin resistance (stratified with the homeostasis model assessment of insulin resistance score (HOMA) of ≥3.8 or <3.8) [33], and infection with hepatitis B virus, were used to determine factors associated with severe fibrosis (F3–F4).

## Results

### Main characteristics of the study population and HIV tropism

We studied 172 HIV–HCV co-infected patients from the ANRS HEPAVIH CO13 cohort: 75 with mild liver fibrosis (F0–F2) and 97 with severe fibrosis (F3–F4). Their median age was 45 years (IQR: 42–48 years) and 133 (77.3%) were males. Most of the patients were former IVDUs (*n* = 128, 76.2%) infected with HCV genotype 1 (*n* = 108, 63.2%). The median duration of HCV infection was 23 years (IQR: 19–27 years) and the median duration of HIV infection was 18 years (IQR: 13–20 years). The majority (91.8%) had received successful antiretroviral therapy (HIV RNA concentration <50 copies/mL in 70.8% of patients). No patient was taking a CCR5 antagonist at inclusion or had already taken a CCR5 antagonist before inclusion into the cohort.

A total of 122 patients (71%) were infected with the R5 virus, 40 with the D/M virus (23%) and 10 with the pure X4 virus (6%). The proportions of CXCR4-using viruses, found by testing the tropism of HIV DNA (24.8%) and HIV RNA (22%), were similar (*p* = 0.60). Because there were so few cases of purely X4 virus infection, the tropism was dichotomized as either a CCR5- or CXCR4-using virus (Table 1).

**Table 1:** Demographic and clinical data for the patients according to their HIV tropism.

	*n*	CCR5-using viruses (*n* = 122)	CXCR4-using viruses (*n* = 50)	*P*-value
**Age**	172	45 (33–65)	44 (35–64)	0.397
**Gender** (male)	172			0.349
Male		92 (75)	41 (82)	
Female		30 (25)	9 (18)	
**Geographic origin**	160			0.50
Europe		81 (71)	34 (74)	
North Africa		23 (20)	8 (17)	
Sub-Saharan Africa		7 (6)	1 (2)	
Other				
**Body-mass index**	167	22.4 (13.9–37.0)	22.0 (17.4–29.4)	0.521
**Insulin resistance**	138			0.767
HOMA <3.8		71 (71)	26 (68)	
HOMA ≥3.8		29 (29)	12 (32)	
**Alcohol consumption**	171			0.988
Never		23 (19)	9 (18)	
Ongoing		43 (36)	18 (36)	
Past		55 (45)	23 (46)	
**Age at infection**				
HCV infection	172	21 (14–43)	21 (13–42)	0.614
HIV infection	171	29 (10–59)	29 (20–46)	0.517
**Duration of infection**				
HCV infection (years)	172	23 (1–42)	23 (1–35)	0.714
HIV infection (years)	171	18 (1–24)	18 (1–23)	0.793
**Source of HCV infection**	162			0.185
IVDU		97 (84)	34 (74)	
Other		19 (16)	12 (26)	
**Source of HIV infection**	171			0.20
IVDU		93 (77)	37 (74)	
Other		27 (23)	13 (26)	
**CDC clinical stage**	172			0.218
A		61 (50)	21 (42)	
B		35 (29)	12 (24)	
C		26 (21)	17 (34)	
**Plasma HIV RNA concentration**	149			0.319
Undetectable (<50 copies/mL)		70 (66)	32 (74)	
Detectable (≥50 copies/mL)		36 (34)	11 (26)	
HIV RNA concentration (when detectable) (log_10_)	47	3.1 (1.7–5.8)	2.8 (1.7–4.3)	0.735
**CD4-cell count at inclusion**	171	403 (48–1878)	342 (55–1327)	0.011
**Nadir CD4-cell count**	153	169 (1–520)	61 (1–405)	<10-4
**ART at inclusion**	172			0.246
Naive		7 (6)	0 (-)	
Pre-treated patients		7 (6)	2 (4)	
Ongoing treatment		108 (88)	48 (96)	
**Class of ART at inclusion**	172			
PI	172	69 (57)	42 (84)	0.001
NNRTI	172	29 (24)	6 (12)	0.082
NRTI	172	103 (84)	46 (92)	0.185
**Cumulative duration under ART before inclusion (months)**	165	116.9 (1.2–233.8)	119.3 (0.9–243.7)	0.908
**Cumulative duration under PI before inclusion (months)**	144	60.1 (1.1–137.9)	59.4 (0.9–199.3)	0.278
**Cumulative duration of NNRTI before inclusion (months)**	109	21.8 (0.3–118.9)	24.3 (0.3–106.3)	0.810
**Cumulative duration of NRTI before inclusion (months)**	165	110.4 (1.2–214.0)	116.5 (0.9–243.7)	0.890
**HCV genotype**	171			0.606
1		74 (61)	34 (70)	
2		2 (2)	0 (-)	
3		25 (20)	10 (20)	
4		21 (17)	5 (10)	
**Plasma HCV RNA concentration** (log_10_; UI/mL)	145	6.2 (3.3–7.8)	6.2 (1.6–7.6)	0.320
**Fibrosis**	172			0.685
F0–F2		52 (43)	23 (46)	
F3–F4		70 (57)	27 (54)	

*Abbreviations*: ART: antiretroviral therapy; CDC: Centers for Disease Control; HOMA: homeostasis model assessment of insulin resistance score; PI: protease inhibitors; NNRTI: non-nucleoside reverse-transcriptase inhibitors; NRTI: nucleoside reverse-transcriptase inhibitors; IQR: inter-quartile range; IVDU: intravenous drug user.

Univariate analyses revealed three factors associated with infection by CXCR4-using viruses: the current CD4-cell count, the nadir CD4-cell count and current treatment with protease inhibitors (Table 2). The only factor associated with the presence of CXCR4-using viruses in multivariate analyses was a nadir of CD4-positive cells <200/mm^3^ (OR: 3.94, 95% CI: 1.39–11.14, *p* = 0.01) (Table 2).

**Table 2:** Factors associated with the presence of CXCR4-using viruses.

	Univariate analyses		Multivariate analyses	
	OR (95%CI)	*P*-value	OR (95%CI)	*P*-value
**Age** (per additional year)	0.89 (0.66–1.21)	0.46	_	
**Gender** (male)	1.48 (0.64–3.41)	0.35	_	
**Age at infection** (per additional year)				
HCV infection	1.04 (0.81–1.32)	0.75	_	
HIV infection	0.91 (0.71–1.15)	0.42	_	
**Duration of infection** (per additional year)				
HCV infection	0.91 (0.72–1.13)	0.38	_	
HIV infection	1.07 (0.79–1.45)	0.66	_	
**Source of HCV infection**				
IVDU vs. other	0.55 (0.24–1.26)	0.16	_	
**Source of HIV infection**				
IVDU vs. other	0.85 (0.40–1.83)	0.69	_	
**CDC clinical stage**				
B vs. A	0.99 (0.43–2.26)	0.99	_	
C vs. A	1.9 (0.86–4.1)	0.11	_	
**Plasma HIV RNA concentration** (≥50 copies/mL)	0.66 (0.30–1.47)	0.32	_	
**CD4-cell count at inclusion** (<200/mm^3^)	2.72 (1.23–6.03)	0.01	_	
**Nadir CD4-cell count** (<200/mm^3^)	5.08 (1.85–13.91)	0.002	3.94 (1.39–11.14)	0.01
**ART at inclusion** (treated vs. untreated-naive)	3.11 (0.68–14.22)	0.14	_	
**Class of ART at inclusion**				
PI	4.03 (1.74–9.31)	0.001	_	
NNRTI	0.43 (0.17–1.13)	0.09	_	
NRTI	2.12 (0.68–6.58)	0.19	_	
**Cumulative duration under ART before inclusion (months)**	1.0 (0.97–1.02)	0.98	_	
**Cumulative duration under PI before inclusion (months)**	1.02 (0.99–1.04)	0.17	_	
**Cumulative duration of NNRTI before inclusion (months)**	1.0 (0.96–1.04)	0.99	_	
**Cumulative duration of NRTI before inclusion (months)**	1.0 (0.98–1.02)	0.94	_	
**HCV genotype**				
2–3 vs. 1–4	0.90 (0.39–2.04)	0.80	_	
**Plasma HCV RNA concentration** (log_10_; IU/mL)	1.11 (0.73–1.70)	0.60	_	

*Abbreviations*: ART: antiretroviral therapy; CDC: Centers for Disease Control; PI: protease inhibitors; NNRTI: non-nucleoside reverse-transcriptase inhibitors; NRTI: nucleoside reverse-transcriptase inhibitors; IQR: inter-quartile range; IVDU: intravenous drug user.

### Plasma HCV RNA

**Figure 1:**
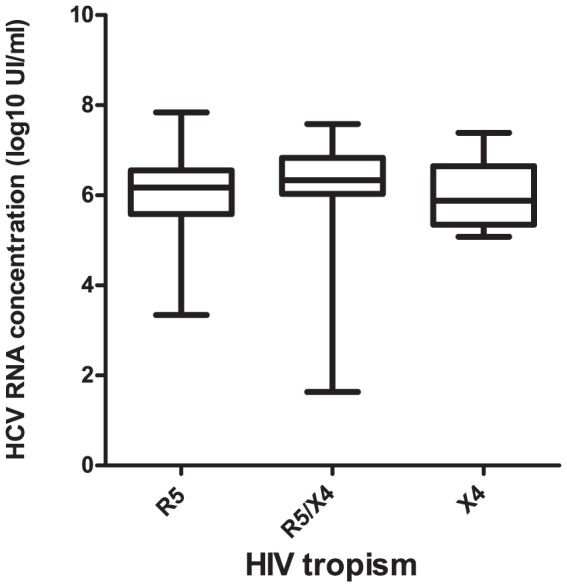
HCV RNA concentrations according to HIV-1 tropism.

We investigated the influence of HIV tropism on the plasma concentration of HCV RNA. The median HCV RNA concentration in patients infected with R5 viruses was 6.2 log_10_ IU/mL (IQR: 3.3–7.8 log_10_ IU/mL); it was 6.3 log_10_ IU/mL (1.6–7.6 log_10_ IU/mL) in patients infected with D/M viruses, and was 5.9 log_10_ IU/mL (5.1–7.4 log_10_ IU/mL) in patients infected with X4 viruses (*p* = 0.28) (Figure 1).

### Predictors of advanced fibrosis

A liver biopsy or clinical signs of cirrhosis indicated that 75 HIV–HCV co-infected patients had mild liver fibrosis (F0–F2) and 97 had severe fibrosis (F3–F4). The proportion of mild fibrosis patients infected with CXCR4-using viruses (*n* = 23, 31%) was similar to that of the severe fibrosis patients (*n* = 27, 28%, *p* = 0.6) (Table 3). Univariate analyses showed several factors associated with severe fibrosis: age, gender, insulin resistance, alcohol consumption, duration of HCV and HIV infections, HCV infection caused by IVDU, current CD4-cell count, nadir CD4-cell count, the duration of exposure to antiretroviral therapy (ART), especially nucleoside reverse-transcriptase inhibitors and steatosis (Table 4).

**Table 3:** Demographic and clinical data for the patients according to liver fibrosis.

	*n*	F0–F2 (*n* = 75)	F3–F4 (*n* = 97)	*P*-value
**Age**	172	43 (33–63)	46 (39–65)	0.005
**Gender** (male)	172			0.003
Male		50 (67)	83 (86)	
Female		25 (33)	14 (14)	
**Geographic origin**	160			0.76
Europe		49 (69)	66 (75)	_
North Africa		14 (20)	17 (19)	_
Sub-Saharan Africa		5 (7)	3 (3)	
Other		3 (4)	3 (3)	
**Body-mass index**	167	22.2 (17.4–32.2)	22.4 (13.9–37.0)	0.967
**Insulin resistance**	138			0.01
HOMA <3.8		49 (82)	48 (62)	
HOMA ≥3.8		11 (18)	30 (38)	
**Alcohol consumption**	171			0.019
Never		17 (23)	15 (16)	
Ongoing		18 (24)	43 (45)	
Past		40 (53)	38 (39)	
**Age at infection**				
HCV infection	172	21 (14–43)	21 (13–43)	0.693
HIV infection	171	28 (10–49)	29 (19–59)	0.506
**Duration of infection**				
HCV infection (years)		22 (1–37)	24 (1–42)	0.004
HIV infection (years)		18 (1–23)	18 (1–24)	0.11
**Source of HCV infection**				0.02
IVDU		55 (73)	76 (87)	
Other		20 (27)	11 (13)	
**Source of HIV infection**	171			0.010
IVDU		49 (66)	81 (84)	
Other		25 (34)	16 (16)	
**CDC clinical stage**	172			0.423
A		40 (53)	42 (43)	
B		18 (24)	29 (30)	
C		17 (23)	26 (27)	
**Plasma HIV RNA concentration**				0.874
Undetectable (<50 copies/mL)		42 (68)	60 (69)	
Detectable (≥50 copies/mL)		20 (32)	27 (31)	
HIV RNA concentration (when detectable) (log_10_)		3.1 (1.8–4.9)	3.1 (1.7–5.8)	0.855
**CD4-cell count at inclusion**	171	396 (55–1878)	356 (48–1185)	0.032
**Nadir CD4-cell count**	153	185 (4–520)	110 (1–480)	0.002
**HIV tropism**	172			0.685
(CCR5-using virus)		52 (69)	70 (72)	
(CXCR4-using virus)		23 (31)	27 (28)	
**ART at inclusion**	172			0.516
Naïve		4 (5)	3 (3)	
Pre-treated patients		5 (7)	4 (4)	
Ongoing treatment		66 (88)	90 (93)	
**Class of ART at inclusion**	172			
PI		47 (63)	64 (66)	0.653
NNRTI		17 (23)	18 (19)	0.507
NRTI		64 (85)	85 (88)	0.661
**Cumulative duration under ART before inclusion (months)**	165	108.1 (0.9–192.1)	120.4 (4.0–243.7)	0.024
**Cumulative duration under PI before inclusion (months)**	144	50.3 (0.9–141.7)	67.6 (1.1–199.3)	0.121
**Cumulative duration of NNRTI before inclusion (months)**	109	28.2 (0.3–118.9)	20.3 (0.3–117.6)	0.724
**Cumulative duration of NRTI before inclusion (months)**	165	105.0 (0.9–192.1)	120.4 (4.0–243.7)	0.027
**HCV genotype**	171			0.064
1		47 (63)	61 (63)	
2		0 (-)	2 (2)	
3		11 (15)	24 (25)	
4		16 (22)	10 (10)	
**Plasma HCV RNA concentration** (log_10_; UI/mL)	145	6.3 (1.6–7.8)	6.1 (3.3–7.8)	0.173
**Steatosis**	112			0.725
≤10%		53 (72)	26 (68)	
>10%		21 (28)	12 (32)	

*Abbreviations*: ART: antiretroviral therapy; CDC: Centers for Disease Control; HOMA: homeostasis model assessment of insulin resistance score; PI: protease inhibitors; NNRTI: non-nucleoside reverse-transcriptase inhibitors; NRTI: nucleoside reverse-transcriptase inhibitors; IQR: inter-quartile range; IVDU: intravenous drug user.

**Table 4:** Factors associated with severe fibrosis (F3–F4).

	Univariate analyses		Multivariate analyses	
	OR (95%CI)	*P*-value	OR (95%CI)	*P*-value
**Age** (per additional year)	1.6 (1.17–2.20)	0.003	1.12 (1.02–1.24)	0.02
**Gender** (male)	2.96 (1.41–6.22)	0.004	_	
**Geographic origin**				
Europe vs. North Africa	1.011 (0.49–2.46)	0.79	_	
Sub-Saharan Africa vs. North Africa	0.49 (0.10–2.44)	0.38	_	
**Body-mass index**	1.001 (0.92–1.10)	0.91	_	
**Insulin resistance** (HOMA ≥3.8)	2.78 (1.25–6.17)	0.012	5.56 (1.29–23.9)	0.02
**Alcohol consumption**		0.02		
Ongoing vs. never	1.07 (0.47–2.45)	0.86	_	
Past vs. never	2.707 (1.11–6.56)	0.02	6.4 (1.26–32.7)	0.02
**Age at infection**				
HCV infection	0.91 (0.72–1.14)	0.42	_	
HIV infection	1.13 (0.91–1.39)	0.26	_	
**Duration of infection**				
HCV infection (≥10 years)	6.47 (1.35–30.9)	0.02	_	
HIV infection (≥10 years)	2.62 (1.08–6.3)	0.03	33.3 (2.21–503.1)	0.01
**Source of HCV infection**				
IVDU vs. other	2.5 (1.11–5.66)	0.02	_	
**Source of HIV infection**				
IVDU vs. other	2.58 (1.25–5.31)	0.01	_	
**CDC clinical stage**				
B vs. A	1.53 (0.73–3.18)	0.25	_	
C vs. A	1.45 (0.68–3.08)	0.32	_	
**Plasma HIV RNA concentration** (≥50 copies/mL)	0.94 (0.46–1.90)	0.87	_	
**CD4 cell-count at inclusion** (<200/mm^3^)	3.68 (1.87–7.25)	<0.01	_	
**Nadir CD4-cell count** (<200/mm^3^)	4.08 (1.94–8.58)	<0.01	17.5 (4.08–74.9)	<0.01
**HIV tropism** (CXCR4-using virus)	0.87 (0.45–1.69)	0.68	_	
**ART at inclusion** (treated vs. untreated)	1.75 (0.62–4.94)	0.29	_	
**Class of ART at inclusion**				
PI	1.15 (0.61–2.16)	0.65	_	
NNRTI	0.77 (0.37–1.63)	0.50	_	
NRTI	1.21 (0.50–2.93)	0.66	_	
**Cumulative duration under ART before inclusion**	1.02 (1.003–1.043)	0.026	_	
**Cumulative duration under PI before inclusion**	1.02 (0.99–1.04)	0.12	_	
**Cumulative duration of NNRTI before inclusion**	0.99 (0.96–1.03)	0.81	_	
**Cumulative duration of NRTI before inclusion**	1.02 (1.00–1.04)	0.028	_	
**HCV genotype**				
1–2–4 vs. 3	0.53 (0.24–1.17)	0.11	_	
**Plasma HCV RNA concentration** (log_10_; UI/mL)	0.83 (0.56–1.23)	0.37	_	
**Steatosis** (>10%)	1.16 (0.49–2.72)	0.72	_	

*Abbreviations*: ART: antiretroviral therapy; CDC: Centers for Disease Control; HOMA: homeostasis model assessment of insulin resistance score; PI: protease inhibitors; NNRTI: non-nucleoside reverse-transcriptase inhibitors; NRTI: nucleoside reverse-transcriptase inhibitors; IQR: inter-quartile range; IVDU: intravenous drug user.

Multivariate analyses identified five factors associated with severe fibrosis (F3–F4): increasing age, insulin resistance (HOMA ≥3.8), past alcohol consumption, duration of HIV infection of ≥10 years and a nadir CD4-cell count of <200/mm^3^ (Table 4).

### Changes in liver stiffness and HIV tropism

We assessed the influence of HIV tropism on the progress of fibrosis in a subgroup of 34 patients. HIV tropism and liver fibrosis, estimated by measuring liver stiffness, were determined at inclusion and at 3 years later. At inclusion, 12/34 were infected with CXCR4-using viruses and 22/34 were infected with CCR5-using viruses. They were all given ART.

**Figure 2:**
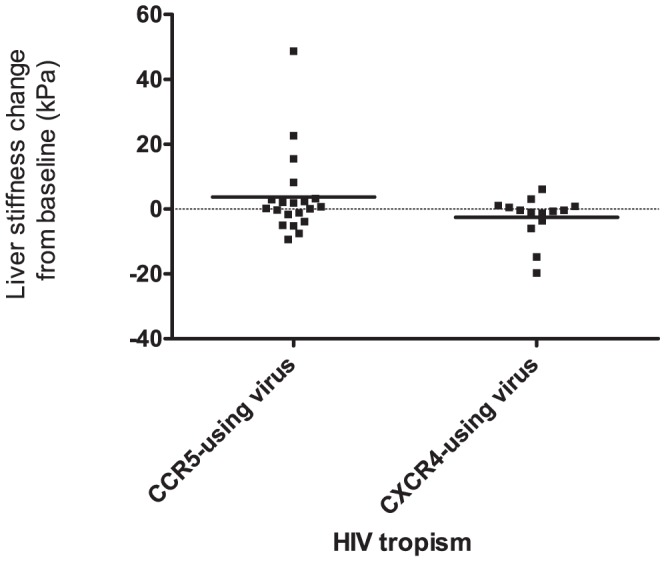
Changes in liver stiffness according to HIV-1 tropism of the 34 patients in the longitudinal study (medians are indicated by bars).

The HIV RNA of 24 (73%) patients was <50 copies/mL throughout the 3-year period. The median HIV RNA concentration in all 34 patients was 2.7 log_10_ copies/mL (IQR: 1.9–3.7 log_10_ copies/mL), it was 3.2 log_10_ copies/mL (IQR: 2.0–4.0 log_10_ copies/mL) in patients with CXCR4-using viruses and 2.5 log_10_ copies/mL (1.9–3.4 log_10_ copies/mL) in patients with CCR5-using viruses (*p* = 0.27). HIV tropism did not evolve in 32 patients, but two patients infected with the R5 virus at inclusion had the R5X4 virus by 36 months later. The median changes in the patients' liver stiffness values relative to baseline values after 36 months were 0.45 kPa (IQR: −2.25, 2.9 kPa) for the 20 patients with a CCR5-using virus and −0.4 kPa (IQR: −0, 0.8 kPa) for the 14 patients with a CXCR4-using virus (*p* = 0.3) (Figure 2).

## Discussion

We assessed the prevalence of CXCR4-using viruses in a population of HIV–HCV co-infected patients and investigated the influence of HIV-1 tropism on plasma concentration of HCV RNA and HCV-related liver fibrosis. The plasma HCV RNA concentrations of patients infected with CCR5-using and CXCR4-using viruses were similar. We also found no relationship between HIV tropism and severe fibrosis in either the cross-sectional study or the longitudinal substudy.

We determined HIV-1 tropism in 172 HIV–HCV co-infected patients at their inclusion into the ANRS CO13 cohort using a phenotypic assay that had been validated for cell-associated HIV-1 DNA or plasma HIV-1 RNA to determine HIV-1 co-receptor usage [32].

Data on HIV tropism from HCV–HIV co-infected subjects are scarce, and there are none on the prevalence of CXCR4-using viruses in this population. A recent study reported that CXCR4-using viruses are more frequently transmitted in HCV-infected IVDUs [31]. CXCR4-using viruses appeared to be less prevalent in our patients compared to highly experienced treatment patients (39–50% of CXCR4-using viruses) [34], [35], [36]. Multivariate analysis indicated that the nadir CD4-cell count was the only factor associated with the presence of CXCR4-using viruses, as previously reported for HIV monoinfected patients [37], [38]. The nadir CD4-cell count was a better marker of HIV disease progression and the presence of CXCR4-using viruses in this population than the current CD4-cell count.

The HCV RNA concentration is higher in HIV–HCV co-infected patients compared to patients with HCV alone [2]. Lin et al. reported that inactivated HIV or recombinant gp120 increased HCV replication *in vitro* [16]. This effect on HCV replication was neutralized by antibodies to CCR5 or CXCR4. This is why we investigated the influence of HIV tropism on HCV RNA concentration *in vivo*. However, our data show that HCV RNA concentrations in patients infected with R5, D/M and X4 viruses were all similar.

We also studied the relationship between HIV tropism and liver fibrosis. Multivariate analyses of the 172 patients in the cross-sectional study identified five factors associated with severe fibrosis: age, alcohol consumption, insulin resistance, the nadir of CD4-cell count and the duration of HIV infection. This supports the current recommendation that these patients limit their alcohol consumption as much as possible. We also found that insulin resistance, estimated using the HOMA score, was associated with liver fibrosis. Insulin resistance has been identified as a factor that promotes steatosis and progression of fibrosis in HCV monoinfected patients [39], [40] and HIV–HCV co-infected patients [41], [42].

It is unclear whether the CD4-cell count or the CD4 nadir is associated with the progression of liver disease. Some have found that lower CD4-cell counts or the CD4 nadir is associated with more severe liver fibrosis and liver outcomes [41], [43], [44], [45], [46], whereas others have found no such association [9], [47]. Nevertheless, HIV-induced immune suppression should be considered a major factor in the progression of liver fibrosis. This supports the recommendation that antiretroviral therapy should be started earlier for HIV–HCV co-infected patients in order to slow down the progression of fibrosis [48].

Having an HIV infection for >10 years is associated with severe fibrosis. However, the estimated durations of infections in the ANRS CO13 cohort differed. The duration of HCV infection was estimated from the year IVDU began, whereas the duration of HIV infection was based on the first positive serological test. This difference may have introduced a bias in the durations of HCV infection. This variable is probably linked to the duration of HCV infection, a major factor associated with the progression of fibrosis [49].

*In vitro* studies have indicated a direct link between HIV tropism and markers for liver fibrogenesis. We postulated that CXCR4-using viruses have a more deleterious impact on liver disease than R5-tropic viruses because of their pro-apoptotic effect on hepatocytes [18], [19] and their stimulation of collagen production by HSCs [17], [20]. However, our cross-sectional study shows that the prevalence of CXCR4-using viruses, in patients with severe fibrosis and in those with mild fibrosis, were similar. The longitudinal follow-up of 34 patients also showed no relationship between HIV tropism and the progression of liver fibrosis. However, the majority of patients in the cross-sectional and longitudinal studies were receiving an antiretroviral therapy. HIV tropism may not affect the progression of fibrosis in patients receiving a potent antiretroviral therapy.

These results agree with those from a recent study by Lin et al. [50], who demonstrated that X4-tropic HIV and R5-tropic HIV trigger the formation of reactive oxygen species in HSC and Huh7.5.1 cells, and that this effect is enhanced by HCV. The production of reactive oxygen species in HSCs, when triggered by HIV, activates profibrogenic genes that encode collagen and the tissue inhibitor metalloprotease-1, as well as down-regulating matrix metallo-protease 3 synthesis. Both X4-tropic HIV and R5-tropic HIV stimulated the production of reactive oxygen species and profibrogenic gene expression to about the same extent [50]. In addition, both X4 and R5 viral infections increased HCV-induced apoptosis of hepatocytes [51]. Therefore, the CXCR4 pathway contributes to liver fibrosis, but the CCR5 pathway has also been demonstrated to play a role in mouse models of liver fibrosis [52]. Moreover, a recent *in vitro* study has demonstrated that a CCR5 antagonist inhibits the migration, proliferation and synthesis of chemokines and collagen secretion by stellate cells in culture [53]. It is perhaps important that a CCR5 antagonist has been shown to greatly ameliorate liver fibrosis in a mouse model [53].

Our study has some limitations. HIV-1 tropism was determined by amplifying HIV DNA from the cells of 145 patients and by amplifying HIV RNA from 27 patients for the cross-sectional study. Nevertheless, a positive correlation has been demonstrated between the abundance of CXCR4-using variants, determined by ultra-deep sequencing in circulating HIV, and the amount of proviral HIV in these cells [54]. As sequential liver biopsies are now rarely taken in clinical practice, we used liver stiffness to estimate changes in liver fibrosis and to assess the influence of HIV tropism on liver fibrosis. This procedure is valid for assessing liver fibrosis in patients with chronic HCV infection, regardless of if it is HIV-negative or HIV-positive [55], [56], [57].

Only a small number of patients were included in our longitudinal study. Yet, the results of this study agree well with those of the cross-sectional study, which supports our conclusion. However, further longitudinal studies on more patients are needed.

In conclusion, we found no relationship between the presence of CXCR4-using viruses and the severity of liver fibrosis or the concentration of HCV RNA in HIV–HCV patients receiving a potent antiretroviral therapy. Recent work suggests that the CCR5 pathway could be antagonized to block fibrosis progression, and further evaluation of the action of CCR5 antagonists in HIV–HCV patients should be assessed.

## Supporting Information

Appendix S1 ANRS CO13 HEPAVIH Study Group.
(DOC)
