# Supplementary material for: HIV-1 Tropism and Liver Fibrosis in HIV–HCV Co-Infected Patients
Source: PLoS One. 2012 Nov 30;7(11):e50289. doi: 10.1371/journal.pone.0050289 (PMC3511493; doi:10.1371/journal.pone.0050289)
Supplement: Appendix S1 — ANRS CO13 HEPAVIH Study Group. (DOC) [file pone.0050289.s001.doc]

**Appendix**

The ANRS CO13 HEPAVIH Study Group is organized as follows:

*Scientific Board:* D. Salmon^1^ (principal investigator), F. Dabis^16^ (principal investigator), M. Winnock^16^, M.A. Loko^16^, P. Sogni^1^, Y. Benhamou^2^, P. Trimoulet^15^, J. Izopet^5^, V. Paradis^2^, B. Spire^17^, P. Carrieri^17^, C. Katlama^2^, G. Pialoux^4^, M.A Valantin^2^, P. Bonnard^4^, I. Poizot-Martin^3^, B. Marchou^5^, E. Rosenthal^6^, A. Bicart-See^8^, R. Djebbar^7^, A. Gervais^9^, C. Lascoux-Combe^10^, C. Goujard^12^, K. Lacombe^11^, C. Duvivier^14^, D. Vittecoq^13^, D. Neau^15^, P. Morlat^15^, F. Bani-Sadr^4^, L. Meyer^12^, F. Boufassa^12^, S. Dominguez^2^, B. Autran^2^, A.M. Roque^13^, C. Solas^3^, H. Fontaine^1^, L. Serfaty^11^, G. Chêne^16^, D. Costagliola^11^, S. Couffin-Cadiergues (ANRS).

*Clinical Centres (ward/participating physicians):*

D. Salmon^1^; P. Sogni^1^; B Terris^1^, Z Makhlouf^1^, G Dubost^1^, F Tessier^1^, L Gibault^1^, F Beuvon^1^, E. Chambon^1^, T. Lazure^1^; A. Krivine^1^; C. Katlama^2^, MA. Valantin^2^, Y. Benhamou^2^; S. Dominguez^2^; F. Charlotte^2^ ; S. Fourati^2^; I. Poizot-Martin^3^, O. Zaegel, A^3^. Ménard^3^; C. Tamalet^3^; G. Pialoux^4^, P. Bonnard^4^, F. Bani-Sadr^4^; P. Callard^4^, F. Bendjaballah^4^; H. Assami^4^; B. Marchou^5^; L.Alric^5^, K. Barange^5^, S. Metivier^5^; Janick Selves^5^; F. Nicot^5^; E. Rosenthal^6^; C. Pradier^6^; J. Haudebourg^6^, M.C Saint-Paul^6^;F. Rouges^7^, R. Djebbar^7^; M. Ziol^7^; Y. Baazia^7^; M. Uzan^8^, A. Bicart-See^8^, D. Garipuy^8^; P. Yéni^9^, A. Gervais^9^; H. Adle-Biassette^9^; D. Séréni^10^, C. Lascoux Combe^10^ P. Bertheau^10^, J. Duclos^10^; P. Palmer^10^; P.M. Girard^11^, K. Lacombe^11^, P. Campa^11^; D. Wendum^11^, P. Cervera^11^, J. Adam^11^; N. Harchi^11^; J.F. Delfraissy^12^, C. Goujard^12^, Y. Quertainmont^12^; C. Pallier^12^;D. Vittecoq^13^; O. Lortholary^14^, C. Duvivier^14^, S. Boucly^14^; D. Neau^15^, P. Morlat^15^, I. Raymond^15^, I. Louis^15^; P. Bioulac-Sage^15^; P. Trimoulet^15^, P. Pinson^15^

*Data collection, management and statistical analyses:* D. Beniken^2^, A-S Ritleng^3^, A. Fooladi, M^5^. Azar^11^, P Honoré^7^, S. Breau^6^, A Joulie^6^, M. Mole^12^, C. Bolliot^13^, F. Touam^14^, F. André^1^, G. Alexandre^9^, A Mélard^14^, J Baume^3^, S. Gillet^16^, J. Delaune^16^, L. Dequae Merchadou^16^, E. Pambrun^16^, A. Frosch^16^, J. Cohen^16^, G Maradan^17^, C Taieb^17^, F Marcellin^17^, M Mora^17^, C Protopopescu^17^, C Lions^17^, MA. Loko^16^, M. Winnock^16^

^1^CHU Cochin, Paris, France

^2^CHU Pitié-Salpétrière, Paris, France

^3^CHU Sainte-Marguerite, Marseille, France

^4^CHU Tenon, Paris, France

^5^CHU Purpan, Toulouse, France

^6^CHU Archet, Nice, France

^7^ CHU Avicenne, Bobigny, France

^8^ Hôpital Joseph-Ducuing, Toulouse, France

^9^ CHU Bichat – Claude-Bernard, Paris, France

^10^ CHU Saint-Louis Paris, France

^11^CHU Saint Antoine Paris, France

^12^ CHU Bicêtre, Paris, France

^13^CHU Paul-Brousse, Villejuif, France

^14^ CHU Necker, Paris, France

^15^ANRS CO 3 Aquitaine cohort, CHU Bordeaux, France

^16^ ISPED, Centre INSERM U897-Epidemiologie-Biostatistique, Bordeaux, France

^17^ INSERM, U912 (SESSTIM), Marseille, France
